# The frequency of Raynaud’s phenomenon, very early diagnosis of systemic sclerosis, and systemic sclerosis in a large Veteran Health Administration database

**DOI:** 10.1186/s41927-021-00209-z

**Published:** 2021-10-15

**Authors:** Tracy M. Frech, Maureen A. Murtaugh, Megan Amuan, Mary Jo Pugh

**Affiliations:** 1 Department of Internal Medicine, Division of Rheumatology, University of Utah and Salt Lake Veterans Affair Medical Center, 1900 E 30 N, SOM 4b200, Salt Lake City, UT 84132 USA; 2 Department of Internal Medicine, University of Utah and Salt Lake Veterans Affair Medical Center, Division of Epidemiology, Salt Lake City, UT USA

**Keywords:** Systemic sclerosis, Scleroderma, Raynaud’s phenomenon, Occupational health

## Abstract

**Background:**

We describe Raynauds phenomenon (RP), potential very early diagnosis of systemic sclerosis (VEDOSS), and systemic sclerosis (SSc) in Veterans deployed in support of Post-9/11 operations. We sought to describe the military occupation specialty, clinical features, and vasodilator use across the three diagnoses.

**Methods:**

Individual Veterans medical records were assessed for RP (ICD-9443.0), VEDOSS with swelling of hands (ICD-9729.81) and RP (ICD-9443.0), and SSc (ICD-9710.1). The distribution of sociodemographic, military service branch, job classification, vasodilator use, and comorbidities were examined across the three classifications of disease. The chi-squared test and Fisher’s exact compared frequency of these categorical variables. Logistic regression assessed the likelihood of characteristics of the three classifications.

**Results:**

In this population of 607,665 individual Veteran medical records, 857 had RP, 45 met possible VEDOSS criteria, and 71 had a diagnosis of SSc. The majority of RP, potential VEDOSS and SSc cases were white males. Those in craftworks, engineering or maintenance, and healthcare had a greater likelihood of RP. Less than half of RP and VEDOSS patients were on vasodilators. The most common comorbidities in this population were the diagnostic code for pain (highest in the potential VEDOSS group [81.6%]), followed by depression in all groups.

**Conclusion:**

This is a unique Veteran population of predominately-male patients. Our data suggests that vasodilator medications are potentially being under-utilized for RP and potential VEDOSS. Our data highlights mood and pain management as an important aspect of SSc care.

## Background

Raynaud’s phenomenon (RP) is a relatively common condition where vasospasm results in discoloration of the fingers and toes from white to blue and then red [1]. RP is classically triggered by cold climate, shifts in temperature, or emotional state. In isolation, without other clinical symptoms, this condition is termed primary RP, and does not have severe consequences. Primary RP is most common in young women, may be familial and can remit over time [2]. Secondary RP is suspected when onset occurs after 40 years of age, it occurs in men, or if attacks are asymmetric, and associated with signs of damage, such as digital ulceration and capillary abnormalities. In addition to behavioral measures, vasodilators are prescribed for management of secondary RP. In patients with RP, the presence of specific antibodies are strongly predictive of development of Systemic Sclerosis (SSc; scleroderma).

SSc is an autoimmune disease characterized by progressive skin and internal multi-organ vasculopathy with resultant fibrosis. In most cases of SSc, the earliest clinical feature is RP [3]. SSc disease onset is commonly defined by first the non-RP manifestation with a longer period between RP and non-RP manifestations in limited cutaneous patients. SSc is most often noted in women as compared to men [4].

An opportunity for early diagnosis and treatment of SSc is properly investigating and treating both RP symptoms and initiating treatment for internal organ involvement [5]. Data suggest that earlier diagnosis may improve outcomes [6]. Specifically, the Very Early Diagnosis of Systemic Sclerosis (VEDOSS) Criteria proactively identifies patients earlier in their disease course so that therapeutics can be initiated prior to end-organ damage [7]. VEDOSS criteria includes RP and puffy fingers, which total five SSc classification criteria points, whereas nine or more points indicate a diagnosis of SSc. Expert guidelines recommend further investigation for a patient with RP and puffy fingers to include laboratory testing for SSc-specific serologies (three classification criteria points) and nailfold capillaroscopy (two classification criteria points). When either of these findings are present in a patient with RP and puffy fingers further diagnostic screens for pulmonary or gastrointestinal tract internal organ involvement are made [8].

Our previous work using Veterans Health Administration (VHA) data in the Veterans Informatics and Computing Infrastructure (VINCI) and natural language processing identified many diagnostic features of SSc among individuals with RP who had not been diagnosed with SSc [9]. The therapeutics most commonly used early in disease for vasculopathy management are vasodilators for RP [10] with immunosuppression generally implemented for fibrotic features. The use of these medications for RP are supported by clinical practice guidelines [11].

The purpose of this project was to assess the prevalence of RP, VEDOSS, and SSc in a cohort of previously deployed Post-9/11 Veterans who received VHA care. We aimed to describe the differential risk of these diagnoses across sociodemographic, specialty military job classification, comorbidities, and relevant medication use in order to inform a thoughtful approach to implementing RP screening and treatment in Veterans. Consistent with prior literature we hypothesized that the prevalence of RP, VEDOSS, and SSc would be significantly higher in women.

## Methods

After Institutional Review Board approval, we identified RP, potential VEDOSS, and SSc, in a cohort of Veterans who were evaluated for long-term outcomes of military exposures. Individuals who a) entered VHA care between October 1, 2001 and September 30, 2011, b) received three or more years of VHA care (inpatient, outpatient, or pharmacy) before September 30, 2014, and c) received at least 1 year of care after 2007 were included. Identification of RP, potential VEDOSS, and SSc using ICD-9-CM codes in VHA clinical care occurred through September 30, 2015.

Similar to our previous work using ICD-9 codes [9], validated by chart abstraction, RP was identified by ICD-9443.0. A potential diagnosis of VEDOSS required a diagnosis of swelling of hands (ICD-9729.81) in addition to a diagnosis of RP (ICD-9443.0). We did not have capillaroscopy and serology data for a definitive diagnosis of VEDOSS. For the purposes of this analysis, a diagnosis of SSc required two separate diagnosis codes (ICD-9710.1) at least 7 days apart. Because these categories may have overlap, we created a hierarchy to develop three mutually exclusive groups. Individuals who met SSc criteria were prioritized, followed by those who met criteria for potential VEDOSS and RP respectively.

Descriptive data included sociodemographic characteristics, military characteristics, and clinical characteristics including vasodilator use, which is the primary treatment for RP. Sociodemographic and military characteristics (Table 1) were defined by the Operation Enduring Freedom/Operation Iraqi Freedom Roster file that was populated by data from the Defense Manpower Data Center. Clinical features/comorbidities were defined using ICD-9 codes in VHA care. Co-morbid conditions that are severity features of SSc, such as esophageal dyskinesia, pulmonary arterial hypertension, interstitial lung disease, and fecal incontinence were examined. Other conditions that can be associated with SSc, such as cancer, liver disease, and kidney disease were included in bivariate analyses.

**Table 1:** Socio-demographics of the study population

	No RP, VEDOSS, or SSc (*n* = 606,692)	Raynauds Phenomenon (*n* = 857)	VEDOSS (*n* = 49)	Systemic Sclerosis (*n* = 71)
Age (years)				
< 29	345,899 (57%)	439 (51%)^a^	14 (31%)	20 (28%)
30–39	137,616 (23%)	213 (25%)^a^	17 (38%)	29 (41%)^a^
40–49	98,582 (16%)	168 (20%)	< 25%	< 25%
50+	24,595 (4%)	37 (4%)	< 5%	< 15%^a^
Married	271,866 (45%)	421 (51%)	25 (56%)	34 (48%)^a^
Unmarried	335,799 (55%)	436 (49%)	20 (45%)	37 (52%)^a^
Sex				
Male	528,329 (87%)	522 (61%)	27 (60%)	41 (58%)
Female	78,363 (13%)	335 (39%)^a^	18 (4 0%)	30 (42%)^a^
Asian	15,878 (3%)	17 (2%)	< 5%	< 5%
Black	107,291 (18%)	165 (19%)	< 20%	22 (31%)^a^
Hispanic	71,375 (12%)	54 (6%)	< 5%	< 15%
Native American	8690 (1%)	< 5%	0	< 10%
White	395,027 (65%)	601 (70%)^a^	35 (78%)	33 (47%)^a^
Unknown	8431 (1%)	< 5%	0	< 5%
Education				
Less than high school	7813 (1%)	11 (1%)	0	0
High School Graduate	46,854 (77%)	589 (69%)	29 (64%)	50 (70%)
Some college	60,347 (10%)	105 (12%)	< 25%	< 15%
College Graduate	46,196 (8%)	97 (11%)	< 15%	< 15%
Post-College	15,650 (3%)	45 (5%)	< 10%	< 5%
Unknown	8144 (1%)	10 (1%)	< 10%	0
Air Force	66,223 (11%)	148 (17%)	10 (22%)	14 (20%)^a^
Coast Guard	77,902 (13%)	102 (12%)	< 10%	< 15%
Marines	82,524 (14%)	71 (8%)	< 10%	< 5%
Army	380,043 (63%)	536 (63%)	29 (64%)	42 (59%)^a^
National Guard/Reserves	245,257 (40%)	303 (35%)	23 (51%)	32 (45%)^a^
Active	361,435 (60%)	554 (65%)	22 (49%)	39 (55%)^a^
Rank				
Warrant/officer	42,877 (7%)	104 (12.%)	< 10%	< 10%
Enlisted	563,815 (93%)	753 (88%)	41 (91%)	65 (92%)^a^
Multiple Deployments				
Yes	319,468 (53%)	438 (51%)	24 (53%)	34 (48%)
No	287,224 (4%)	419 (49%)	21 (47%)	37 (52%)
Job Category				
Administrative	79,973 (14%)	147 (17%)	< 20%	14 (20%)
Craftworkers	41,036 (7%)	74 (9%)	< 10%	< 10%
Intelligence	44,980 (7%)	59 (7%)	< 10%	< 10%
Health Care	27,009 (1%)	77 (9%)^a^	< 15%	< 10%
Tactical Operations	13,490 (2%)	14 (2%)	< 10%	0
Infantry	143,653 (24%)	133 (16%)	< 15%	< 15%
Supply	102,614 (17%)	132 (15%)	< 20%	12 (17%)
Allied Specialist	11,470 (2%)	21 (2%)	0	< 10%
Electronic Repairs	126,193 (21%)	164 (19%)	< 25%	19 (27%)
Engineering/	6869 (1%)	18 (2%)^a^	< 10%	< 10%
Maintenance				
Unknown	9425 (2%)	18 (2%)	< 10%	< 10%

^a^denotes statistical significance across the categories from no conditions, to RP, VEDOSS and SSc

Comparison across study groups used the chi-squared test. Three multivariable logistic regression analyses compared SSc, potential VEDOSS, and RP groups to those with no indicators. Due to the low number of cases, only pulmonary arterial hypertension, hypertension and cancer were included in the multivariable models. Analyses were conducted with SAS® V 9.3.

## Results

In this cohort (*N* = 606,692), 857 had RP (0.14%), 45 met potential VEDOSS criteria (0.01%), and 71 (0.01%) had a diagnosis of SSc (Table 1). The median age of patients with RP were under 29 years of age, where as potential VEDOSS and SSc patients were age 30–39. The SSc cases occurred more frequently in white males. RP (*n* = 536, 63%), potential VEDOSS (*n* = 29, 64%), and SSc cases (*n* = 42, 59%) were more likely to have prior Army service.

Fewer than half of those with RP/potential VEDOSS and 63% of SSc patients received vasodilator prescriptions (Table 2). Pain related diagnoses (back pain, neck pain, other musculoskeletal/neuropathic pain) were common (highest in the potential VEDOSS group [81.6%]), as was depression in all groups. Suicidal ideation/attempt was greater than 5% in all groups. While raw numbers of SSc-specific comorbidities were low in each group, of interest these SSc clinical features were also reported in RP patients, which, if studied longitudinally, may help in an understanding in the pathogenic link between vasculopathy and end organ damage (Table 2). In multivariable logistic regression models (Table 3), women and those less than 50 years of age had consistently higher odds of each condition compared to those with no conditions. RP patients were more likely to be health care, engineering /maintenance or craft-workers. Veterans with RP were more likely to have pulmonary artery hypertension (adjusted odds ratio [AOR] 5.0; 95% confidence interval [CI]: 2.1–12.2), hypertension (AOR = 1.6; 95% CI: 1.4–1.9) and cancer (AOR = 1.8; 95% CI: 1.2–2.7) were associated an odds ratio of 1.8 for RP versus controls.

**Table 2:** Clinical Features

	No RP, VEDOSS, or SSc (*n* = 606,692)	Raynauds Phenomenon (*n* = 857)	VEDOSS (*n* = 49)	Systemic Sclerosis (*n* = 71)
Vasodilator use	92,530 (15%)	410 (48%)	21 (47%)	45 (63%)
Pain	228,045 (38%)	493 (58%)	37 (82%)	46 (65%)
Fecal incontinence	829 (0.1%)	< 1%	< 5%	< 5%
Esophageal Dyskinesia	564 (0.1%)	< 1%	0	< 10%^a^
Interstitial Lung Disease	11 (0%)	0	0	< 5%^a^
Pulmonary Arterial Hypertension	542 (0.1%)	< 1%	0	15 (21%)^a^
Cancer	8736 (1%)	27 (3%)	0	< 5%
High Blood Pressure	120,368 (20%)	222 (26%)^a^	13 (29%)	33 (47%)^a^
Diabetes	29,780 (5%)	34 (4%)	< 5%	< 10%
Anxiety	145,477 (24%)	269 (31%)	11 (24%)	21 (30%)
Heart Disease	19,297 (3%)	50 (6%)	< 15%	< 15%
Liver Disease	9740 (2%)	14 (2%)	0	< 10%
Kidney Disease	4545 (1%)	18 (2%)	0	< 5%
Suicidal ideation/attempt	32,050 (5%)	59 (7%)	< 10%	< 10%
Obesity	122,838 (20%)	134 (16%)	< 25%	12 (17%)
Seizures	8821 (2%)	21 (2%)	< 5%	< 5%
Depression	219,302 (36%)	384 (45%)^a^	22 (49%)^a^	39 (55%)^a^
PTSD	257,801 (42%)	394 (46%)	22 (49%)	26 (37%)

^a^denotes statistical significance across the categories from no conditions, RP, VEDOSS and SSc

**Table 3:** Demographic, Clinical Features and Military Occupation Characteristics Associated with Raynauds Phenomenon, Very Early Diagnosis of Systemic Sclerosis, and Systemic Sclerosis

	Raynauds Phenomenon (*n* = 857)	VEDOSS (*n* = 49)	Systemic Sclerosis (*n* = 71)
Age (yrs)			
< 29	1.0	1.0	1.0
30–39	1.2 (.9–1.4)	3.2 (1.5–6.5)*	3.0 (1.7–5.5)*
40–49	1.2 (1.2–1.5)*	2.9 (1.3–6.6)*	1.9 0.9–3.8)*
50+	0.9 (0.7–1.3)	2.9 (0.8–10.6)	3.3 (1.3–8.4)*
Sex			
Male	1.0	1.0	1.0
Female	4.8 (4.1–5.9)*	5.9 (3.2–11.0)*	5.8 (3.4–9.8)*
Race			
White	1.0	1.0	1.0
All others	1.5 (1.3–1.8)*	2.6 (1.3–5.4)*	0.7 (0.4–1.1)
Army	1.0	1.0	1.0
Other Armed Forces	1.0 (0.9–1.2)	1.1 (0.6–1.9)	0.8 (0.5–2.0)
Traumatic Brain Injury	0.9 (0.8–1.1)	0.8 (0.4–1.7)	1.01 (0.5–2.0)
Pulmonary Artery Hypertension	5.0 (2.1–12.2)*	–	–
Hypertension	1.6 (1.4–1.9)*	1.6 (0.8–3.1)	3.0 (1.8–5)*
Cancer	1.8 (1.2–2.7)*	–	1.7 (0.6–05.9)
Job			
Craftworker	1.3 (1.0–1.8)*	–	1.2 (0.4–3.5)
Electronic Equipment Worker	1.1 (0.8–1.3)	–	1.6 (0.8–3.4)
Engineering/Maintenance	1.8 (1.1–2.9)*	–	2.3 (0.5–10.2)
Health Care	1.5 (1.2–3.0)*	–	0.9 (0.3–2.7)
Infantry	1.0 (0.7–1.2)	–	1.0 (0.4–2.7)
Intelligence	1.0 (0.8–1.4)	–	1.34 (0.5–3.8)
Technical/Allied Specialist	1.3 (0.8–2.1)	–	0.9 (0.1–6.7)
Supply	0.9 (0.7–1.1)	–	1.0 (0.5–2.3)
Tactical Operations	0.9 (0.5–1.5)	–	–

* 95% confidence Interval, statistically significant (*p* < 0.05) compared to the group no conditions

## Discussion

This is the first reported evaluation of RP, potential VEDOSS, and SSc in this relatively young cohort of Post-9/11 Veterans. This provides a unique population of SSc patients that can allow a better understanding of the natural history of this connective tissue disease characterized by vasculopathy and progressive fibrosis in several respects. It allows study in a primarily male population, provides insight into possible military exposures associated with RP, describes associated comorbidities, and possible treatment gaps. Our study suggests an under-diagnosis of these conditions in Veterans and the need for better screening and treatment of vasculopathy.

This study found that in a this health system, RP, VEDOSS and SSc occur possibly at a higher rate in men in the VA than the general population. The increased likelihood of RP, potential VEDOSS and SSc in Veteran females is consistent with estimates from other population-based studies of SSc and RP [12–14]. The study of a predominately-male cohort is important since there are more severe internal organ-based complications and higher mortality in men with SSc [15]. Our previous work using Veteran’s electronic health records highlights that ICD-9 code may not capture all cases of SSc in Veterans [9].

The younger age of those with RP than with VEDOSS or SSc supports the concept of reporting both RP and non-RP disease duration in analyses of SSc is important for understanding pathogenesis [16]. In this present study, we identified individuals with VEDOSS, patients with puffy hands and RP who are at higher risk for SSc, but who do not yet meet full criteria for SSc. An increase prevalence of autoimmune disorders and musculoskeletal pain is reported among Veterans serving in Afghanistan and Iraq [17–19]. While our study does not adequately characterize the source of pain, our data suggest that there is a need for further surveillance of Veterans with RP and VEDOSS to understand disease features and potentially that SSc is under-diagnosed in male Veterans.

There was no difference in the likelihood RP, potential VEDOSS or SSc across branch of military service. This result should be verified in future studies due to the rare nature of VEDOSS and SSc. However, RP patients were more likely to be craft-workers, health care workers, or in engineering /maintenance. Perhaps RP symptoms are more commonly reported in Veterans using their fingers for fine motor activities.

Alternatively, this may highlight the value of understanding occupational exposures that are unique to this patient population. As an example, polyvinyl chloride is used largely as coatings for computer cables and wires and is reported as a potential occupational risk factor for SSc [20–23]. Exposure to cleaning products, solvents and adhesives is associated with increased risk of SSc [24]. Exposures to these products could be greater among craft-workers, health care workers, and those in engineering/maintenance. Our work highlights the importance of further exposure assessments in all service branches. There is evidence in other military cohorts that exposures are important for autoantibody formation in autoimmune disease [25]. Our study emphasizes that increased awareness and improved protective professional measures are indicated. Differences in SSc risk between males and females may exist [26], and this cohort may allow future research to further examine those differences.

Use of vasodilators is standard of care for management of secondary RP, VEDOSS and SSc. We were unable to differentiate between primary and secondary RP in this cohort but our data suggests that vasodilators are potentially being under-utilized in VEDOSS and SSc Veteran population. Vasodilators treat both RP and hypertension. The importance of blood pressure monitoring and identification of hypertension is important in SSc patients [27]. This is a potentially unmet management need in the Veteran population. Our data demonstrates high prevalence of depression consistent with other reports that highlight mood and pain management is an important aspect of SSc [28]. Our data suggests this is also true for RP and potential VEDOSS patients. This cohort provides the possible opportunity for improved management of Veterans with RP, VEDOSS, and SSc.

The use of a national cohort of Veterans is a strength; however, there are also some limitations. In this cohort, the low prevalence of RP suggests that perhaps this billing code is underutilized. The prevalence of SSc in this cohort is also low compared to the prevalence reported in the literature, which could be a result of missed cases through the ICD-9 code identification that could have led to selection bias. As such, the small number of patients included in the potential VEDOSS and SSc sub-cohorts limit robust conclusions. The predominance of young males in the examined cohorts limits the extraction of any general conclusions for SSc and certain disease features such as esophageal involvement and pulmonary fibrosis. We did not have information on primary versus secondary RP. We did not have information on exposures associated with RP, such as use of vibration tools or amphetamines. In order to reduce errors associated with “rule out” diagnoses, identification of RP, potential VEDOSS and SSc cases required two diagnoses at least 7 days apart, which is likely a conservative estimate, especially for SSc cases where we did not have access to many clinical signs or biomarkers for SSc, such as ANA or autoantibody data, and subtype characterization of limited versus diffuse cutaneous disease. Our characterization of potential VEDOSS would be strengthened by serology data and capillaroscopy reports. The severity of RP and etiology of pain may introduce bias and explain the low use of vasodilators. Further classification of pain as a diagnostic code is needed to best interpret this symptom. Moreover, data from care in non-VA health care systems, including specialty care for SSc, is not represented in our database.

## Conclusions

This study highlights the importance of further prospective research on RP, potential VEDOSS, and SSc in the unique Military and Veteran populations that includes assessment of environmental exposures, comorbidity identification, and use of vasodilators. There is a large number of men with RP, potential VEDOSS and SSc that suggests that the DOD and VAMC system will be very useful in examining both the natural history of SSc among Veterans. This unique cohort can allow understanding of phenotypic differences in VEDOSS and SSc by sex and possible service duty exposures.

## Data Availability

The military records used to datasets generated and/or analysed during the current study are not publicly available, but are available from the authors on reasonable request.

## Abbreviations

SSc: Systemic sclerosis
RP: Raynaud’s phenomenon
VEDOSS: Very Early Diagnosis of Systemic Sclerosis
ICD: International Classification of Diseases
DOD: Department of Defense
VAMC: Veterans Affair Medical Center
VHA: Veterans Health Administration
VINCI: Veterans Informatics and Computing Infrastructure
